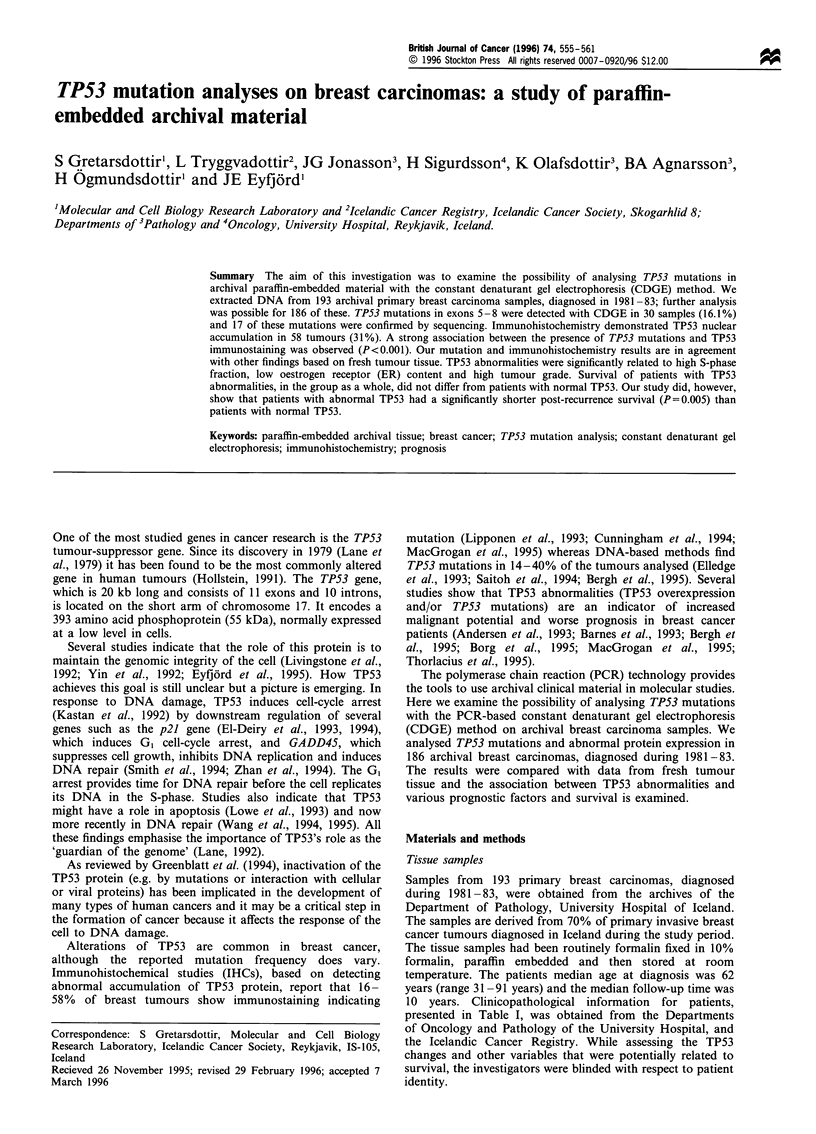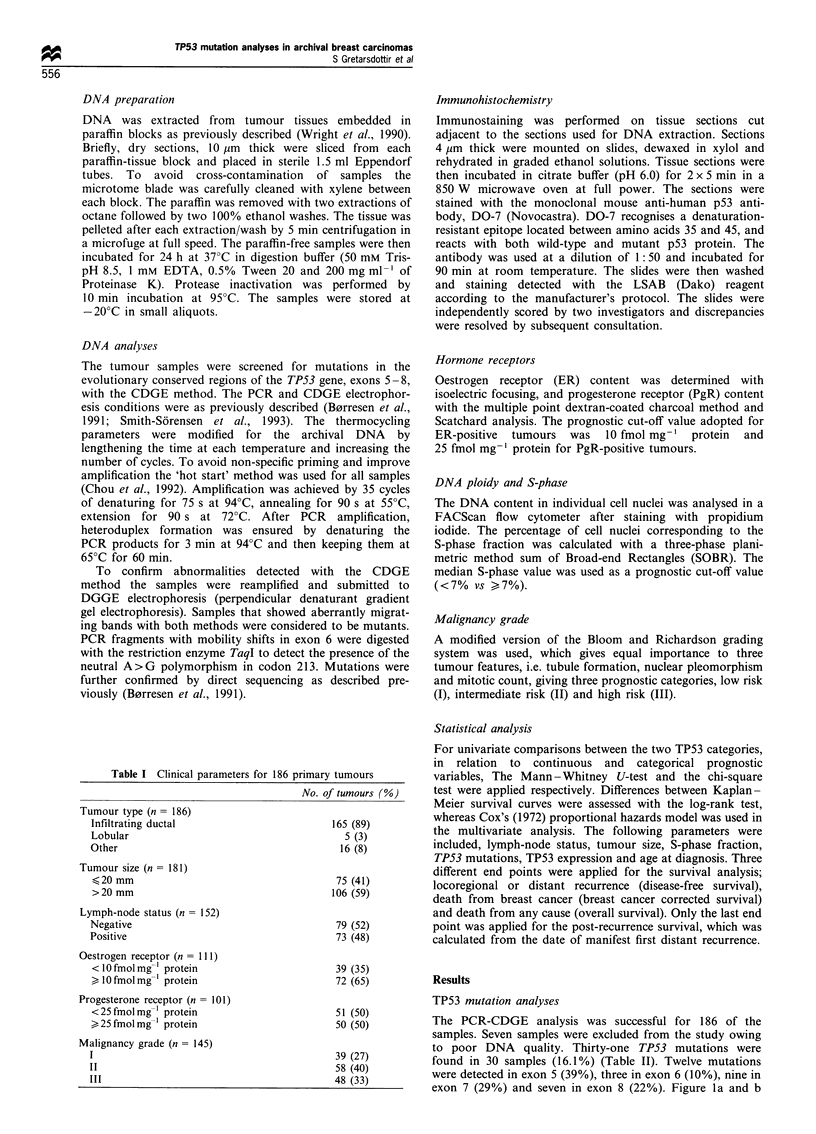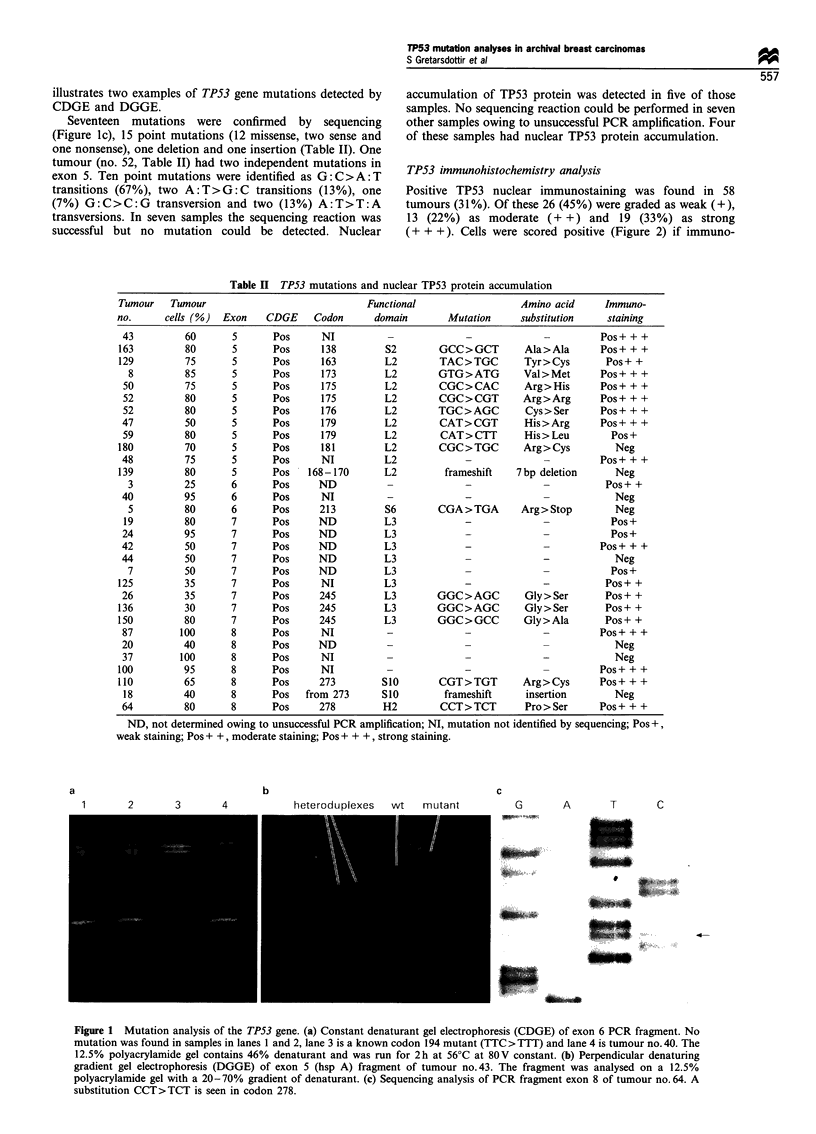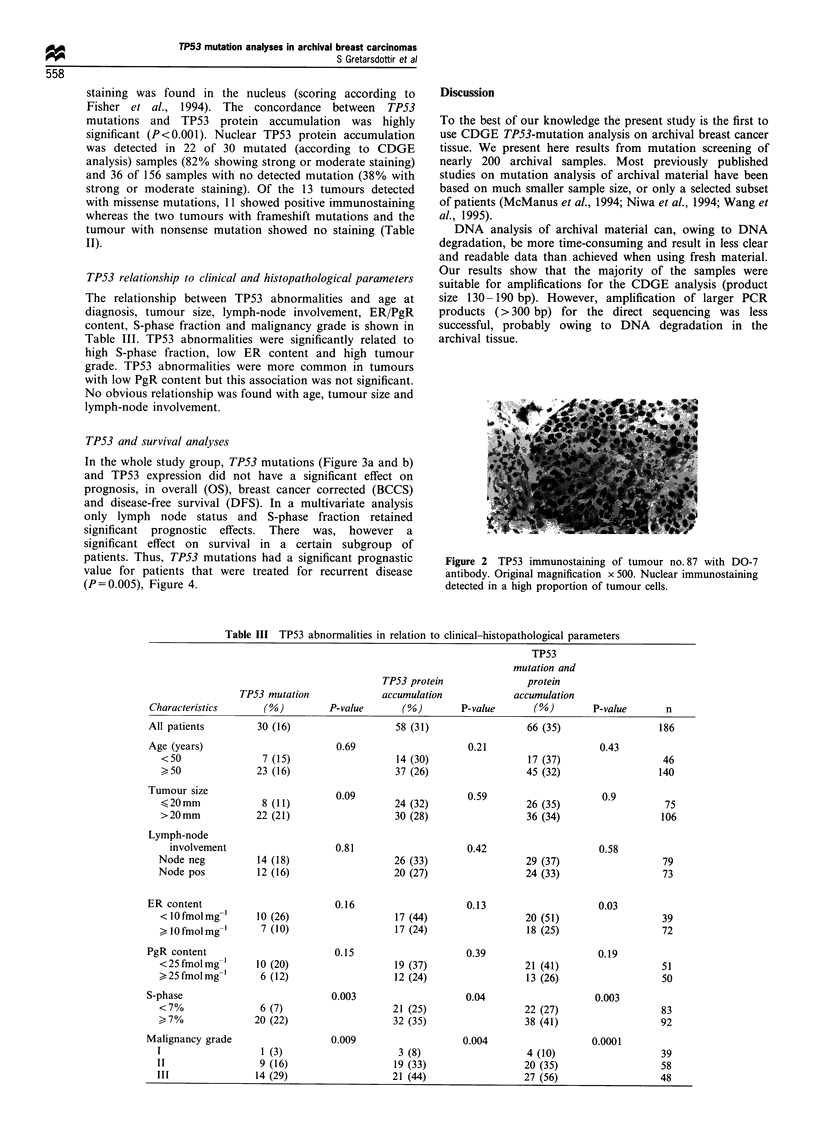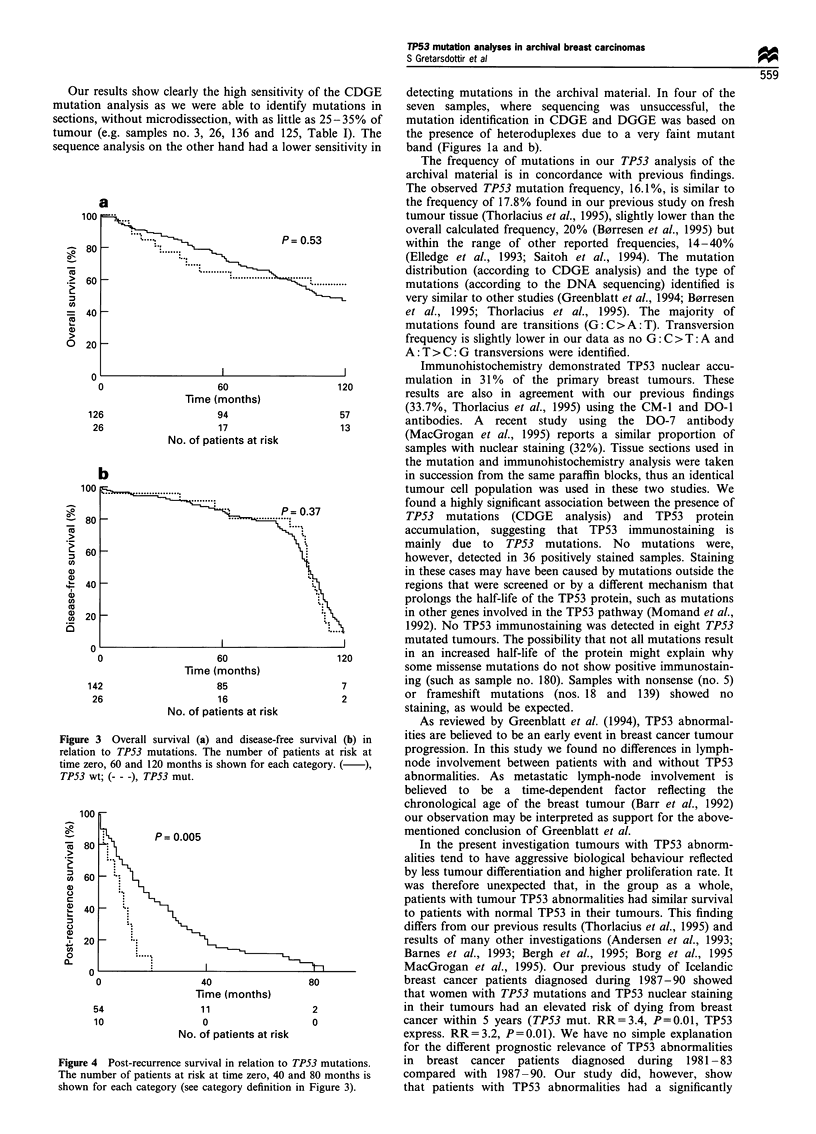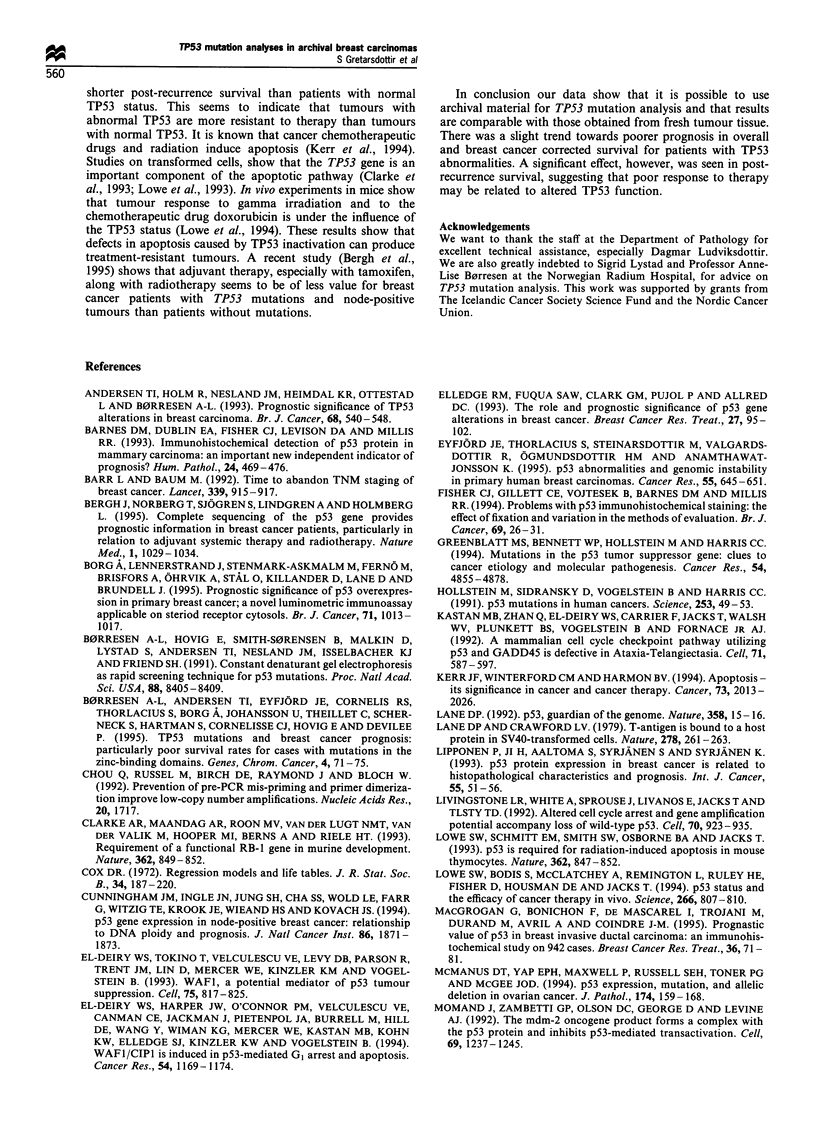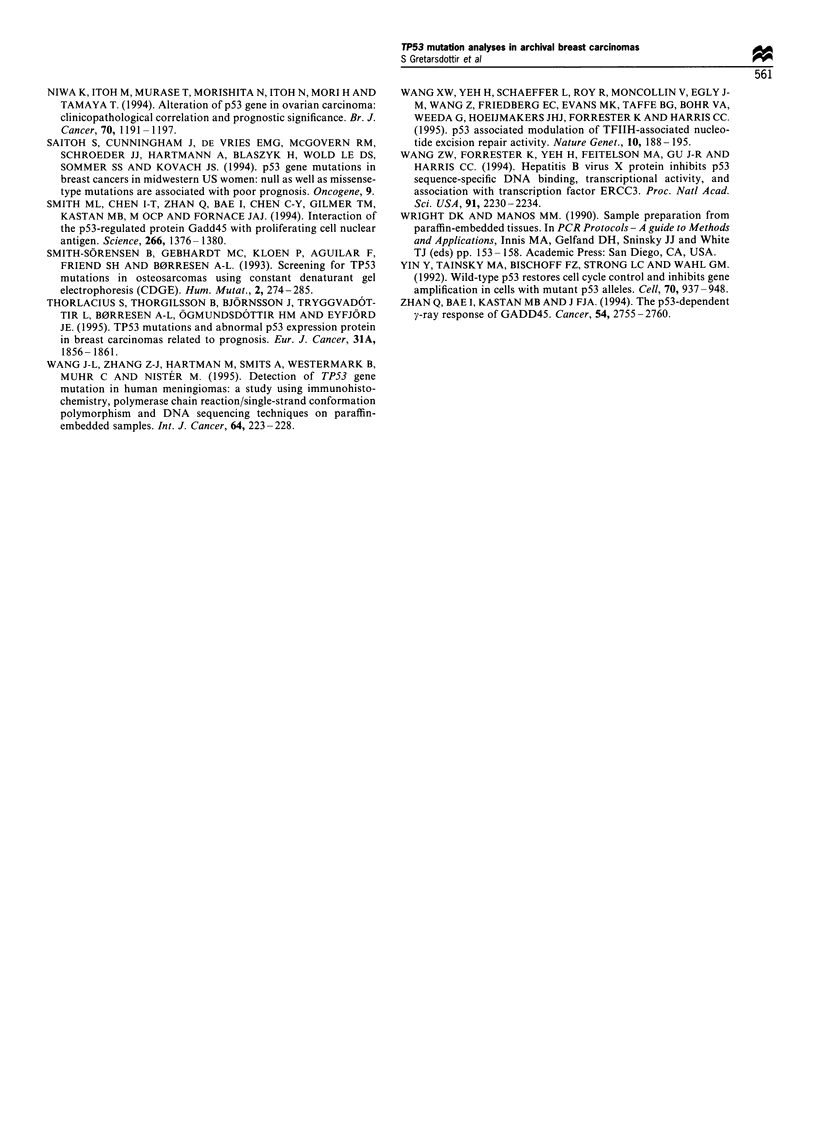# TP53 mutation analyses on breast carcinomas: a study of paraffin-embedded archival material.

**DOI:** 10.1038/bjc.1996.400

**Published:** 1996-08

**Authors:** S. Gretarsdottir, L. Tryggvadottir, J. G. Jonasson, H. Sigurdsson, K. Olafsdottir, B. A. Agnarsson, H. Ogmundsdottir, J. E. Eyfjörd

**Affiliations:** Molecular and Cell Biology Research Laboratory, Icelandic Cancer Society, Reykjavik, Iceland.

## Abstract

**Images:**


British Journal of Cancer (1996) 74, 555-561
© 1996 Stockton Press All rights reserved 0007-0920/96 $12.00

### *TP53* mutation analyses on breast carcinomas: a study of paraffin-embedded archival material

S Gretarsdottir[1], L Tryggvadottir[2], JG Jonasson[3], H Sigurdsson[4], K Olafsdottir[3], BA Agnarsson[3], H Ögmundsdottir[1] and JE Eyfjörd[1]

*[1]Molecular and Cell Biology Research Laboratory and [2]Icelandic Cancer Registry, Icelandic Cancer Society, Skogarhlid 8; Departments of [3]Pathology and [4]Oncology, University Hospital, Reykjavik, Iceland.*

**Summary** The aim of this investigation was to examine the possibility of analysing *TP53* mutations in archival paraffin-embedded material with the constant denaturant gel electrophoresis (CDGE) method. We extracted DNA from 193 archival primary breast carcinoma samples, diagnosed in 1981–83; further analysis was possible for 186 of these. *TP53* mutations in exons 5–8 were detected with CDGE in 30 samples (16.1%) and 17 of these mutations were confirmed by sequencing. Immunohistochemistry demonstrated TP53 nuclear accumulation in 58 tumours (31%). A strong association between the presence of *TP53* mutations and TP53 immunostaining was observed ($P < 0.001$). Our mutation and immunohistochemistry results are in agreement with other findings based on fresh tumour tissue. TP53 abnormalities were significantly related to high S-phase fraction, low oestrogen receptor (ER) content and high tumour grade. Survival of patients with TP53 abnormalities, in the group as a whole, did not differ from patients with normal TP53. Our study did, however, show that patients with abnormal TP53 had a significantly shorter post-recurrence survival ($P = 0.005$) than patients with normal TP53.

**Keywords:** paraffin-embedded archival tissue; breast cancer; *TP53* mutation analysis; constant denaturant gel electrophoresis; immunohistochemistry; prognosis

One of the most studied genes in cancer research is the *TP53* tumour-suppressor gene. Since its discovery in 1979 (Lane *et al.*, 1979) it has been found to be the most commonly altered gene in human tumours (Hollstein, 1991). The *TP53* gene, which is 20 kb long and consists of 11 exons and 10 introns, is located on the short arm of chromosome 17. It encodes a 393 amino acid phosphoprotein (55 kDa), normally expressed at a low level in cells.

Several studies indicate that the role of this protein is to maintain the genomic integrity of the cell (Livingstone *et al.*, 1992; Yin *et al.*, 1992; Eyfjörd *et al.*, 1995). How TP53 achieves this goal is still unclear but a picture is emerging. In response to DNA damage, TP53 induces cell-cycle arrest (Kastan *et al.*, 1992) by downstream regulation of several genes such as the *p21* gene (El-Deiry *et al.*, 1993, 1994), which induces $G_1$ cell-cycle arrest, and *GADD45*, which suppresses cell growth, inhibits DNA replication and induces DNA repair (Smith *et al.*, 1994; Zhan *et al.*, 1994). The $G_1$ arrest provides time for DNA repair before the cell replicates its DNA in the S-phase. Studies also indicate that TP53 might have a role in apoptosis (Lowe *et al.*, 1993) and now more recently in DNA repair (Wang *et al.*, 1994, 1995). All these findings emphasise the importance of TP53's role as the 'guardian of the genome' (Lane, 1992).

As reviewed by Greenblatt *et al.* (1994), inactivation of the TP53 protein (e.g. by mutations or interaction with cellular or viral proteins) has been implicated in the development of many types of human cancers and it may be a critical step in the formation of cancer because it affects the response of the cell to DNA damage.

Alterations of TP53 are common in breast cancer, although the reported mutation frequency does vary. Immunohistochemical studies (IHCs), based on detecting abnormal accumulation of TP53 protein, report that 16–58% of breast tumours show immunostaining indicating mutation (Lipponen *et al.*, 1993; Cunningham *et al.*, 1994; MacGrogan *et al.*, 1995) whereas DNA-based methods find *TP53* mutations in 14–40% of the tumours analysed (Elledge *et al.*, 1993; Saitoh *et al.*, 1994; Bergh *et al.*, 1995). Several studies show that TP53 abnormalities (TP53 overexpression and/or *TP53* mutations) are an indicator of increased malignant potential and worse prognosis in breast cancer patients (Andersen *et al.*, 1993; Barnes *et al.*, 1993; Bergh *et al.*, 1995; Borg *et al.*, 1995; MacGrogan *et al.*, 1995; Thorlacius *et al.*, 1995).

The polymerase chain reaction (PCR) technology provides the tools to use archival clinical material in molecular studies. Here we examine the possibility of analysing *TP53* mutations with the PCR-based constant denaturant gel electrophoresis (CDGE) method on archival breast carcinoma samples. We analysed *TP53* mutations and abnormal protein expression in 186 archival breast carcinomas, diagnosed during 1981–83. The results were compared with data from fresh tumour tissue and the association between TP53 abnormalities and various prognostic factors and survival is examined.

### Materials and methods

### *Tissue samples*

Samples from 193 primary breast carcinomas, diagnosed during 1981–83, were obtained from the archives of the Department of Pathology, University Hospital of Iceland. The samples are derived from 70% of primary invasive breast cancer tumours diagnosed in Iceland during the study period. The tissue samples had been routinely formalin fixed in 10% formalin, paraffin embedded and then stored at room temperature. The patients median age at diagnosis was 62 years (range 31–91 years) and the median follow-up time was 10 years. Clinicopathological information for patients, presented in Table I, was obtained from the Departments of Oncology and Pathology of the University Hospital, and the Icelandic Cancer Registry. While assessing the TP53 changes and other variables that were potentially related to survival, the investigators were blinded with respect to patient identity.

Correspondence: S Gretarsdottir, Molecular and Cell Biology Research Laboratory, Icelandic Cancer Society, Reykjavik, IS-105, Iceland
Recieved 26 November 1995; revised 29 February 1996; accepted 7 March 1996

*DNA preparation*

DNA was extracted from tumour tissues embedded in paraffin blocks as previously described (Wright *et al.*, 1990). Briefly, dry sections, 10 μm thick were sliced from each paraffin-tissue block and placed in sterile 1.5 ml Eppendorf tubes. To avoid cross-contamination of samples the microtome blade was carefully cleaned with xylene between each block. The paraffin was removed with two extractions of octane followed by two 100% ethanol washes. The tissue was pelleted after each extraction/wash by 5 min centrifugation in a microfuge at full speed. The paraffin-free samples were then incubated for 24 h at 37°C in digestion buffer (50 mM Tris-pH 8.5, 1 mM EDTA, 0.5% Tween 20 and 200 mg $ml^{-1}$ of Proteinase K). Protease inactivation was performed by 10 min incubation at 95°C. The samples were stored at −20°C in small aliquots.

*DNA analyses*

The tumour samples were screened for mutations in the evolutionary conserved regions of the *TP53* gene, exons 5–8, with the CDGE method. The PCR and CDGE electrophoresis conditions were as previously described (Børresen *et al.*, 1991; Smith-Sörensen *et al.*, 1993). The thermocycling parameters were modified for the archival DNA by lengthening the time at each temperature and increasing the number of cycles. To avoid non-specific priming and improve amplification the 'hot start' method was used for all samples (Chou *et al.*, 1992). Amplification was achieved by 35 cycles of denaturing for 75 s at 94°C, annealing for 90 s at 55°C, extension for 90 s at 72°C. After PCR amplification, heteroduplex formation was ensured by denaturing the PCR products for 3 min at 94°C and then keeping them at 65°C for 60 min.

To confirm abnormalities detected with the CDGE method the samples were reamplified and submitted to DGGE electrophoresis (perpendicular denaturant gradient gel electrophoresis). Samples that showed aberrantly migrating bands with both methods were considered to be mutants. PCR fragments with mobility shifts in exon 6 were digested with the restriction enzyme *Taq*I to detect the presence of the neutral A>G polymorphism in codon 213. Mutations were further confirmed by direct sequencing as described previously (Børresen *et al.*, 1991).

**Table I** Clinical parameters for 186 primary tumours

| | *No. of tumours (%)* |
|---|---|
| Tumour type (*n* = 186) | |
| Infiltrating ductal | 165 (89) |
| Lobular | 5 (3) |
| Other | 16 (8) |
| Tumour size (*n* = 181) | |
| ⩽20 mm | 75 (41) |
| >20 mm | 106 (59) |
| Lymph-node status (*n* = 152) | |
| Negative | 79 (52) |
| Positive | 73 (48) |
| Oestrogen receptor (*n* = 111) | |
| <10 fmol $mg^{-1}$ protein | 39 (35) |
| ⩾10 fmol $mg^{-1}$ protein | 72 (65) |
| Progesterone receptor (*n* = 101) | |
| <25 fmol $mg^{-1}$ protein | 51 (50) |
| ⩾25 fmol $mg^{-1}$ protein | 50 (50) |
| Malignancy grade (*n* = 145) | |
| I | 39 (27) |
| II | 58 (40) |
| III | 48 (33) |

*Immunohistochemistry*

Immunostaining was performed on tissue sections cut adjacent to the sections used for DNA extraction. Sections 4 μm thick were mounted on slides, dewaxed in xylol and rehydrated in graded ethanol solutions. Tissue sections were then incubated in citrate buffer (pH 6.0) for 2 × 5 min in a 850 W microwave oven at full power. The sections were stained with the monoclonal mouse anti-human p53 antibody, DO-7 (Novocastra). DO-7 recognises a denaturation-resistant epitope located between amino acids 35 and 45, and reacts with both wild-type and mutant p53 protein. The antibody was used at a dilution of 1 : 50 and incubated for 90 min at room temperature. The slides were then washed and staining detected with the LSAB (Dako) reagent according to the manufacturer's protocol. The slides were independently scored by two investigators and discrepancies were resolved by subsequent consultation.

*Hormone receptors*

Oestrogen receptor (ER) content was determined with isoelectric focusing, and progesterone receptor (PgR) content with the multiple point dextran-coated charcoal method and Scatchard analysis. The prognostic cut-off value adopted for ER-positive tumours was 10 fmol $mg^{-1}$ protein and 25 fmol $mg^{-1}$ protein for PgR-positive tumours.

*DNA ploidy and S-phase*

The DNA content in individual cell nuclei was analysed in a FACScan flow cytometer after staining with propidium iodide. The percentage of cell nuclei corresponding to the S-phase fraction was calculated with a three-phase planimetric method sum of Broad-end Rectangles (SOBR). The median S-phase value was used as a prognostic cut-off value (<7% *vs* ⩾7%).

*Malignancy grade*

A modified version of the Bloom and Richardson grading system was used, which gives equal importance to three tumour features, i.e. tubule formation, nuclear pleomorphism and mitotic count, giving three prognostic categories, low risk (I), intermediate risk (II) and high risk (III).

*Statistical analysis*

For univariate comparisons between the two TP53 categories, in relation to continuous and categorical prognostic variables, The Mann–Whitney *U*-test and the chi-square test were applied respectively. Differences between Kaplan–Meier survival curves were assessed with the log-rank test, whereas Cox's (1972) proportional hazards model was used in the multivariate analysis. The following parameters were included, lymph-node status, tumour size, S-phase fraction, *TP53* mutations, TP53 expression and age at diagnosis. Three different end points were applied for the survival analysis; locoregional or distant recurrence (disease-free survival), death from breast cancer (breast cancer corrected survival) and death from any cause (overall survival). Only the last end point was applied for the post-recurrence survival, which was calculated from the date of manifest first distant recurrence.

### Results

TP53 *mutation analyses*

The PCR-CDGE analysis was successful for 186 of the samples. Seven samples were excluded from the study owing to poor DNA quality. Thirty-one *TP53* mutations were found in 30 samples (16.1%) (Table II). Twelve mutations were detected in exon 5 (39%), three in exon 6 (10%), nine in exon 7 (29%) and seven in exon 8 (22%). Figure 1a and b illustrates two examples of *TP53* gene mutations detected by CDGE and DGGE.

Seventeen mutations were confirmed by sequencing (Figure 1c), 15 point mutations (12 missense, two sense and one nonsense), one deletion and one insertion (Table II). One tumour (no. 52, Table II) had two independent mutations in exon 5. Ten point mutations were identified as G:C>A:T transitions (67%), two A:T>G:C transitions (13%), one (7%) G:C>C:G transversion and two (13%) A:T>T:A transversions. In seven samples the sequencing reaction was successful but no mutation could be detected. Nuclear accumulation of TP53 protein was detected in five of those samples. No sequencing reaction could be performed in seven other samples owing to unsuccessful PCR amplification. Four of these samples had nuclear TP53 protein accumulation.

### *TP53 immunohistochemistry analysis*

Positive TP53 nuclear immunostaining was found in 58 tumours (31%). Of these 26 (45%) were graded as weak (+), 13 (22%) as moderate (+ +) and 19 (33%) as strong (+ + +). Cells were scored positive (Figure 2) if immuno-

**Table II** *TP53* mutations and nuclear TP53 protein accumulation

| *Tumour no.* | *Tumour cells (%)* | *Exon* | *CDGE* | *Codon* | *Functional domain* | *Mutation* | *Amino acid substitution* | *Immuno-staining* |
|---|---|---|---|---|---|---|---|---|
| 43 | 60 | 5 | Pos | NI | – | – | – | Pos+ + + |
| 163 | 80 | 5 | Pos | 138 | S2 | GCC>GCT | Ala>Ala | Pos+ + + |
| 129 | 75 | 5 | Pos | 163 | L2 | TAC>TGC | Tyr>Cys | Pos+ + |
| 8 | 85 | 5 | Pos | 173 | L2 | GTG>ATG | Val>Met | Pos+ + + |
| 50 | 75 | 5 | Pos | 175 | L2 | CGC>CAC | Arg>His | Pos+ + + |
| 52 | 80 | 5 | Pos | 175 | L2 | CGC>CGT | Arg>Arg | Pos+ + + |
| 52 | 80 | 5 | Pos | 176 | L2 | TGC>AGC | Cys>Ser | Pos+ + + |
| 47 | 50 | 5 | Pos | 179 | L2 | CAT>CGT | His>Arg | Pos+ + + |
| 59 | 80 | 5 | Pos | 179 | L2 | CAT>CTT | His>Leu | Pos+ |
| 180 | 70 | 5 | Pos | 181 | L2 | CGC>TGC | Arg>Cys | Neg |
| 48 | 75 | 5 | Pos | NI | L2 | – | – | Pos+ + + |
| 139 | 80 | 5 | Pos | 168–170 | L2 | frameshift | 7 bp deletion | Neg |
| 3 | 25 | 6 | Pos | ND | – | – | – | Pos+ + |
| 40 | 95 | 6 | Pos | NI | – | – | – | Neg |
| 5 | 80 | 6 | Pos | 213 | S6 | CGA>TGA | Arg>Stop | Neg |
| 19 | 80 | 7 | Pos | ND | L3 | – | – | Pos+ |
| 24 | 95 | 7 | Pos | ND | L3 | – | – | Pos+ |
| 42 | 50 | 7 | Pos | ND | L3 | – | – | Pos+ + + |
| 44 | 50 | 7 | Pos | ND | L3 | – | – | Neg |
| 7 | 50 | 7 | Pos | ND | L3 | – | – | Pos+ |
| 125 | 35 | 7 | Pos | NI | L3 | – | – | Pos+ + |
| 26 | 35 | 7 | Pos | 245 | L3 | GGC>AGC | Gly>Ser | Pos+ + |
| 136 | 30 | 7 | Pos | 245 | L3 | GGC>AGC | Gly>Ser | Pos+ + |
| 150 | 80 | 7 | Pos | 245 | L3 | GGC>GCC | Gly>Ala | Pos+ + |
| 87 | 100 | 8 | Pos | NI | – | – | – | Pos+ + + |
| 20 | 40 | 8 | Pos | ND | – | – | – | Neg |
| 37 | 100 | 8 | Pos | NI | – | – | – | Neg |
| 100 | 95 | 8 | Pos | NI | – | – | – | Pos+ + + |
| 110 | 65 | 8 | Pos | 273 | S10 | CGT>TGT | Arg>Cys | Pos+ + + |
| 18 | 40 | 8 | Pos | from 273 | S10 | frameshift | insertion | Neg |
| 64 | 80 | 8 | Pos | 278 | H2 | CCT>TCT | Pro>Ser | Pos+ + + |

ND, not determined owing to unsuccessful PCR amplification; NI, mutation not identified by sequencing; Pos+, weak staining; Pos+ +, moderate staining; Pos+ + +, strong staining.

**Figure 1** Mutation analysis of the *TP53* gene. (**a**) Constant denaturant gel electrophoresis (CDGE) of exon 6 PCR fragment. No mutation was found in samples in lanes 1 and 2, lane 3 is a known codon 194 mutant (TTC>TTT) and lane 4 is tumour no. 40. The 12.5% polyacrylamide gel contains 46% denaturant and was run for 2 h at 56°C at 80 V constant. (**b**) Perpendicular denaturing gradient gel electrophoresis (DGGE) of exon 5 (hsp A) fragment of tumour no. 43. The fragment was analysed on a 12.5% polyacrylamide gel with a 20–70% gradient of denaturant. (**c**) Sequencing analysis of PCR fragment exon 8 of tumour no. 64. A substitution CCT>TCT is seen in codon 278.

staining was found in the nucleus (scoring according to Fisher *et al*., 1994). The concordance between *TP53* mutations and TP53 protein accumulation was highly significant ($P<0.001$). Nuclear TP53 protein accumulation was detected in 22 of 30 mutated (according to CDGE analysis) samples (82% showing strong or moderate staining) and 36 of 156 samples with no detected mutation (38% with strong or moderate staining). Of the 13 tumours detected with missense mutations, 11 showed positive immunostaining whereas the two tumours with frameshift mutations and the tumour with nonsense mutation showed no staining (Table II).

### *TP53 relationship to clinical and histopathological parameters*

The relationship between TP53 abnormalities and age at diagnosis, tumour size, lymph-node involvement, ER/PgR content, S-phase fraction and malignancy grade is shown in Table III. TP53 abnormalities were significantly related to high S-phase fraction, low ER content and high tumour grade. TP53 abnormalities were more common in tumours with low PgR content but this association was not significant. No obvious relationship was found with age, tumour size and lymph-node involvement.

### *TP53 and survival analyses*

In the whole study group, *TP53* mutations (Figure 3a and b) and TP53 expression did not have a significant effect on prognosis, in overall (OS), breast cancer corrected (BCCS) and disease-free survival (DFS). In a multivariate analysis only lymph node status and S-phase fraction retained significant prognostic effects. There was, however a significant effect on survival in a certain subgroup of patients. Thus, *TP53* mutations had a significant prognastic value for patients that were treated for recurrent disease ($P=0.005$), Figure 4.

### Discussion

To the best of our knowledge the present study is the first to use CDGE *TP53*-mutation analysis on archival breast cancer tissue. We present here results from mutation screening of nearly 200 archival samples. Most previously published studies on mutation analysis of archival material have been based on much smaller sample size, or only a selected subset of patients (McManus *et al*., 1994; Niwa *et al*., 1994; Wang *et al*., 1995).

DNA analysis of archival material can, owing to DNA degradation, be more time-consuming and result in less clear and readable data than achieved when using fresh material. Our results show that the majority of the samples were suitable for amplifications for the CDGE analysis (product size 130–190 bp). However, amplification of larger PCR products (>300 bp) for the direct sequencing was less successful, probably owing to DNA degradation in the archival tissue.

**Figure 2** TP53 immunostaining of tumour no. 87 with DO-7 antibody. Original magnification ×500. Nuclear immunostaining detected in a high proportion of tumour cells.

**Table III** TP53 abnormalities in relation to clinical–histopathological parameters

| *Characteristics* | *TP53 mutation (%)* | *P-value* | *TP53 protein accumulation (%)* | P-*value* | TP53 *mutation and protein accumulation (%)* | P-*value* | n |
|---|---|---|---|---|---|---|---|
| All patients | 30 (16) | | 58 (31) | | 66 (35) | | 186 |
| Age (years) | | 0.69 | | 0.21 | | 0.43 | |
| <50 | 7 (15) | | 14 (30) | | 17 (37) | | 46 |
| ⩾50 | 23 (16) | | 37 (26) | | 45 (32) | | 140 |
| Tumour size | | 0.09 | | 0.59 | | 0.9 | |
| ⩽20 mm | 8 (11) | | 24 (32) | | 26 (35) | | 75 |
| >20 mm | 22 (21) | | 30 (28) | | 36 (34) | | 106 |
| Lymph-node involvement | | 0.81 | | 0.42 | | 0.58 | |
| Node neg | 14 (18) | | 26 (33) | | 29 (37) | | 79 |
| Node pos | 12 (16) | | 20 (27) | | 24 (33) | | 73 |
| ER content | | 0.16 | | 0.13 | | 0.03 | |
| <10 fmol $mg^{-1}$ | 10 (26) | | 17 (44) | | 20 (51) | | 39 |
| ⩾10 fmol $mg^{-1}$ | 7 (10) | | 17 (24) | | 18 (25) | | 72 |
| PgR content | | 0.15 | | 0.39 | | 0.19 | |
| <25 fmol $mg^{-1}$ | 10 (20) | | 19 (37) | | 21 (41) | | 51 |
| ⩾25 fmol $mg^{-1}$ | 6 (12) | | 12 (24) | | 13 (26) | | 50 |
| S-phase | | 0.003 | | 0.04 | | 0.003 | |
| <7% | 6 (7) | | 21 (25) | | 22 (27) | | 83 |
| ⩾7% | 20 (22) | | 32 (35) | | 38 (41) | | 92 |
| Malignancy grade | | 0.009 | | 0.004 | | 0.0001 | |
| I | 1 (3) | | 3 (8) | | 4 (10) | | 39 |
| II | 9 (16) | | 19 (33) | | 20 (35) | | 58 |
| III | 14 (29) | | 21 (44) | | 27 (56) | | 48 |

Our results show clearly the high sensitivity of the CDGE mutation analysis as we were able to identify mutations in sections, without microdissection, with as little as 25–35% of tumour (e.g. samples no. 3, 26, 136 and 125, Table I). The sequence analysis on the other hand had a lower sensitivity in

**Figure 3** Overall survival (**a**) and disease-free survival (**b**) in relation to *TP53* mutations. The number of patients at risk at time zero, 60 and 120 months is shown for each category. (——), *TP53* wt; (- - -), *TP53* mut.

**Figure 4** Post-recurrence survival in relation to *TP53* mutations. The number of patients at risk at time zero, 40 and 80 months is shown for each category (see category definition in Figure 3).

detecting mutations in the archival material. In four of the seven samples, where sequencing was unsuccessful, the mutation identification in CDGE and DGGE was based on the presence of heteroduplexes due to a very faint mutant band (Figures 1a and b).

The frequency of mutations in our *TP53* analysis of the archival material is in concordance with previous findings. The observed *TP53* mutation frequency, 16.1%, is similar to the frequency of 17.8% found in our previous study on fresh tumour tissue (Thorlacius *et al*., 1995), slightly lower than the overall calculated frequency, 20% (Børresen *et al*., 1995) but within the range of other reported frequencies, 14–40% (Elledge *et al*., 1993; Saitoh *et al*., 1994). The mutation distribution (according to CDGE analysis) and the type of mutations (according to the DNA sequencing) identified is very similar to other studies (Greenblatt *et al*., 1994; Børresen *et al*., 1995; Thorlacius *et al*., 1995). The majority of mutations found are transitions (G:C>A:T). Transversion frequency is slightly lower in our data as no G:C>T:A and A:T>C:G transversions were identified.

Immunohistochemistry demonstrated TP53 nuclear accumulation in 31% of the primary breast tumours. These results are also in agreement with our previous findings (33.7%, Thorlacius *et al*., 1995) using the CM-1 and DO-1 antibodies. A recent study using the DO-7 antibody (MacGrogan *et al*., 1995) reports a similar proportion of samples with nuclear staining (32%). Tissue sections used in the mutation and immunohistochemistry analysis were taken in succession from the same paraffin blocks, thus an identical tumour cell population was used in these two studies. We found a highly significant association between the presence of *TP53* mutations (CDGE analysis) and TP53 protein accumulation, suggesting that TP53 immunostaining is mainly due to *TP53* mutations. No mutations were, however, detected in 36 positively stained samples. Staining in these cases may have been caused by mutations outside the regions that were screened or by a different mechanism that prolongs the half-life of the TP53 protein, such as mutations in other genes involved in the TP53 pathway (Momand *et al*., 1992). No TP53 immunostaining was detected in eight *TP53* mutated tumours. The possibility that not all mutations result in an increased half-life of the protein might explain why some missense mutations do not show positive immunostaining (such as sample no. 180). Samples with nonsense (no. 5) or frameshift mutations (nos. 18 and 139) showed no staining, as would be expected.

As reviewed by Greenblatt *et al*. (1994), TP53 abnormalities are believed to be an early event in breast cancer tumour progression. In this study we found no differences in lymph-node involvement between patients with and without TP53 abnormalities. As metastatic lymph-node involvement is believed to be a time-dependent factor reflecting the chronological age of the breast tumour (Barr *et al*., 1992) our observation may be interpreted as support for the above-mentioned conclusion of Greenblatt *et al*.

In the present investigation tumours with TP53 abnormalities tend to have aggressive biological behaviour reflected by less tumour differentiation and higher proliferation rate. It was therefore unexpected that, in the group as a whole, patients with tumour TP53 abnormalities had similar survival to patients with normal TP53 in their tumours. This finding differs from our previous results (Thorlacius *et al*., 1995) and results of many other investigations (Andersen *et al*., 1993; Barnes *et al*., 1993; Bergh *et al*., 1995; Borg *et al*., 1995 MacGrogan *et al*., 1995). Our previous study of Icelandic breast cancer patients diagnosed during 1987–90 showed that women with *TP53* mutations and TP53 nuclear staining in their tumours had an elevated risk of dying from breast cancer within 5 years (*TP53* mut. RR = 3.4, $P = 0.01$, TP53 express. RR = 3.2, $P = 0.01$). We have no simple explanation for the different prognostic relevance of TP53 abnormalities in breast cancer patients diagnosed during 1981–83 compared with 1987–90. Our study did, however, show that patients with TP53 abnormalities had a significantly shorter post-recurrence survival than patients with normal TP53 status. This seems to indicate that tumours with abnormal TP53 are more resistant to therapy than tumours with normal TP53. It is known that cancer chemotherapeutic drugs and radiation induce apoptosis (Kerr *et al.*, 1994). Studies on transformed cells, show that the *TP53* gene is an important component of the apoptotic pathway (Clarke *et al.*, 1993; Lowe *et al.*, 1993). *In vivo* experiments in mice show that tumour response to gamma irradiation and to the chemotherapeutic drug doxorubicin is under the influence of the TP53 status (Lowe *et al.*, 1994). These results show that defects in apoptosis caused by TP53 inactivation can produce treatment-resistant tumours. A recent study (Bergh *et al.*, 1995) shows that adjuvant therapy, especially with tamoxifen, along with radiotherapy seems to be of less value for breast cancer patients with *TP53* mutations and node-positive tumours than patients without mutations.

In conclusion our data show that it is possible to use archival material for *TP53* mutation analysis and that results are comparable with those obtained from fresh tumour tissue. There was a slight trend towards poorer prognosis in overall and breast cancer corrected survival for patients with TP53 abnormalities. A significant effect, however, was seen in post-recurrence survival, suggesting that poor response to therapy may be related to altered TP53 function.

**Acknowledgements**
We want to thank the staff at the Department of Pathology for excellent technical assistance, especially Dagmar Ludviksdottir. We are also greatly indebted to Sigrid Lystad and Professor Anne-Lise Børresen at the Norwegian Radium Hospital, for advice on *TP53* mutation analysis. This work was supported by grants from The Icelandic Cancer Society Science Fund and the Nordic Cancer Union.

### References

ANDERSEN TI, HOLM R, NESLAND JM, HEIMDAL KR, OTTESTAD L AND BØRRESEN A-L. (1993). Prognostic significance of TP53 alterations in breast carcinoma. *Br. J. Cancer*, **68**, 540–548.

BARNES DM, DUBLIN EA, FISHER CJ, LEVISON DA AND MILLIS RR. (1993). Immunohistochemical detection of p53 protein in mammary carcinoma: an important new independent indicator of prognosis? *Hum. Pathol.*, **24**, 469–476.

BARR L AND BAUM M. (1992). Time to abandon TNM staging of breast cancer. *Lancet*, **339**, 915–917.

BERGH J, NORBERG T, SJÖGREN S, LINDGREN A AND HOLMBERG L. (1995). Complete sequencing of the p53 gene provides prognostic information in breast cancer patients, particularly in relation to adjuvant systemic therapy and radiotherapy. *Nature Med.*, **1**, 1029–1034.

BORG Å, LENNERSTRAND J, STENMARK-ASKMALM M, FERNÖ M, BRISFORS A, ÖHRVIK A, STÅL O, KILLANDER D, LANE D AND BRUNDELL J. (1995). Prognostic significance of p53 overexpression in primary breast cancer; a novel luminometric immunoassay applicable on steriod receptor cytosols. *Br. J. Cancer*, **71**, 1013–1017.

BØRRESEN A-L, HOVIG E, SMITH-SØRENSEN B, MALKIN D, LYSTAD S, ANDERSEN TI, NESLAND JM, ISSELBACHER KJ AND FRIEND SH. (1991). Constant denaturant gel electrophoresis as rapid screening technique for p53 mutations. *Proc. Natl Acad. Sci. USA*, **88**, 8405–8409.

BØRRESEN A-L, ANDERSEN TI, EYFJÖRD JE, CORNELIS RS, THORLACIUS S, BORG Å, JOHANSSON U, THEILLET C, SCHERNECK S, HARTMAN S, CORNELISSE CJ, HOVIG E AND DEVILEE P. (1995). TP53 mutations and breast cancer prognosis: particularly poor survival rates for cases with mutations in the zinc-binding domains. *Genes, Chrom. Cancer*, **4**, 71–75.

CHOU Q, RUSSEL M, BIRCH DE, RAYMOND J AND BLOCH W. (1992). Prevention of pre-PCR mis-priming and primer dimerization improve low-copy number amplifications. *Nucleic Acids Res.*, **20**, 1717.

CLARKE AR, MAANDAG AR, ROON MV, VAN DER LUGT NMT, VAN DER VALIK M, HOOPER MI, BERNS A AND RIELE HT. (1993). Requirement of a functional RB-1 gene in murine development. *Nature*, **362**, 849–852.

COX DR. (1972). Regression models and life tables. *J. R. Stat. Soc. B.*, **34**, 187–220.

CUNNINGHAM JM, INGLE JN, JUNG SH, CHA SS, WOLD LE, FARR G, WITZIG TE, KROOK JE, WIEAND HS AND KOVACH JS. (1994). p53 gene expression in node-positive breast cancer: relationship to DNA ploidy and prognosis. *J. Natl Cancer Inst.* **86**, 1871–1873.

EL-DEIRY WS, TOKINO T, VELCULESCU VE, LEVY DB, PARSON R, TRENT JM, LIN D, MERCER WE, KINZLER KM AND VOGELSTEIN B. (1993). WAF1, a potential mediator of p53 tumour suppression. *Cell*, **75**, 817–825.

EL-DEIRY WS, HARPER JW, O'CONNOR PM, VELCULESCU VE, CANMAN CE, JACKMAN J, PIETENPOL JA, BURRELL M, HILL DE, WANG Y, WIMAN KG, MERCER WE, KASTAN MB, KOHN KW, ELLEDGE SJ, KINZLER KW AND VOGELSTEIN B. (1994). WAF1/CIP1 is induced in p53-mediated $G_1$ arrest and apoptosis. *Cancer Res.*, **54**, 1169–1174.

ELLEDGE RM, FUQUA SAW, CLARK GM, PUJOL P AND ALLRED DC. (1993). The role and prognostic significance of p53 gene alterations in breast cancer. *Breast Cancer Res. Treat.*, **27**, 95–102.

EYFJÖRD JE, THORLACIUS S, STEINARSDOTTIR M, VALGARDSDOTTIR R, ÖGMUNDSDOTTIR HM AND ANAMTHAWAT-JONSSON K. (1995). p53 abnormalities and genomic instability in primary human breast carcinomas. *Cancer Res.*, **55**, 645–651.

FISHER CJ, GILLETT CE, VOJTESEK B, BARNES DM AND MILLIS RR. (1994). Problems with p53 immunohistochemical staining: the effect of fixation and variation in the methods of evaluation. *Br. J. Cancer*, **69**, 26–31.

GREENBLATT MS, BENNETT WP, HOLLSTEIN M AND HARRIS CC. (1994). Mutations in the p53 tumor suppressor gene: clues to cancer etiology and molecular pathogenesis. *Cancer Res.*, **54**, 4855–4878.

HOLLSTEIN M, SIDRANSKY D, VOGELSTEIN B AND HARRIS CC. (1991). p53 mutations in human cancers. *Science*, **253**, 49–53.

KASTAN MB, ZHAN Q, EL-DEIRY WS, CARRIER F, JACKS T, WALSH WV, PLUNKETT BS, VOGELSTEIN B AND FORNACE JR AJ. (1992). A mammalian cell cycle checkpoint pathway utilizing p53 and GADD45 is defective in Ataxia-Telangiectasia. *Cell*, **71**, 587–597.

KERR JF, WINTERFORD CM AND HARMON BV. (1994). Apoptosis – its significance in cancer and cancer therapy. *Cancer*, **73**, 2013–2026.

LANE DP. (1992). p53, guardian of the genome. *Nature*, **358**, 15–16.

LANE DP AND CRAWFORD LV. (1979). T-antigen is bound to a host protein in SV40-transformed cells. *Nature*, **278**, 261–263.

LIPPONEN P, JI H, AALTOMA S, SYRJÄNEN S AND SYRJÄNEN K. (1993). p53 protein expression in breast cancer is related to histopathological characteristics and prognosis. *Int. J. Cancer*, **55**, 51–56.

LIVINGSTONE LR, WHITE A, SPROUSE J, LIVANOS E, JACKS T AND TLSTY TD. (1992). Altered cell cycle arrest and gene amplification potential accompany loss of wild-type p53. *Cell*, **70**, 923–935.

LOWE SW, SCHMITT EM, SMITH SW, OSBORNE BA AND JACKS T. (1993). p53 is required for radiation-induced apoptosis in mouse thymocytes. *Nature*, **362**, 847–852.

LOWE SW, BODIS S, MCCLATCHEY A, REMINGTON L, RULEY HE, FISHER D, HOUSMAN DE AND JACKS T. (1994). p53 status and the efficacy of cancer therapy in vivo. *Science*, **266**, 807–810.

MACGROGAN G, BONICHON F, DE MASCAREL I, TROJANI M, DURAND M, AVRIL A AND COINDRE J-M. (1995). Prognastic value of p53 in breast invasive ductal carcinoma: an immunohistochemical study on 942 cases. *Breast Cancer Res. Treat.*, **36**, 71–81.

MCMANUS DT, YAP EPH, MAXWELL P, RUSSELL SEH, TONER PG AND MCGEE JOD. (1994). p53 expression, mutation, and allelic deletion in ovarian cancer. *J. Pathol.*, **174**, 159–168.

MOMAND J, ZAMBETTI GP, OLSON DC, GEORGE D AND LEVINE AJ. (1992). The mdm-2 oncogene product forms a complex with the p53 protein and inhibits p53-mediated transactivation. *Cell*, **69**, 1237–1245.

NIWA K, ITOH M, MURASE T, MORISHITA N, ITOH N, MORI H AND TAMAYA T. (1994). Alteration of p53 gene in ovarian carcinoma: clinicopathological correlation and prognostic significance. *Br. J. Cancer*, **70,** 1191–1197.

SAITOH S, CUNNINGHAM J, DE VRIES EMG, MCGOVERN RM, SCHROEDER JJ, HARTMANN A, BLASZYK H, WOLD LE DS, SOMMER SS AND KOVACH JS. (1994). p53 gene mutations in breast cancers in midwestern US women: null as well as missense-type mutations are associated with poor prognosis. *Oncogene*, **9**.

SMITH ML, CHEN I-T, ZHAN Q, BAE I, CHEN C-Y, GILMER TM, KASTAN MB, M OCP AND FORNACE JAJ. (1994). Interaction of the p53-regulated protein Gadd45 with proliferating cell nuclear antigen. *Science*, **266,** 1376–1380.

SMITH-SÖRENSEN B, GEBHARDT MC, KLOEN P, AGUILAR F, FRIEND SH AND BØRRESEN A-L. (1993). Screening for TP53 mutations in osteosarcomas using constant denaturant gel electrophoresis (CDGE). *Hum. Mutat.*, **2,** 274–285.

THORLACIUS S, THORGILSSON B, BJÖRNSSON J, TRYGGVADÓTTIR L, BØRRESEN A-L, ÖGMUNDSDÓTTIR HM AND EYFJÖRD JE. (1995). TP53 mutations and abnormal p53 expression protein in breast carcinomas related to prognosis. *Eur. J. Cancer*, **31A,** 1856–1861.

WANG J-L, ZHANG Z-J, HARTMAN M, SMITS A, WESTERMARK B, MUHR C AND NISTÉR M. (1995). Detection of *TP53* gene mutation in human meningiomas: a study using immunohistochemistry, polymerase chain reaction/single-strand conformation polymorphism and DNA sequencing techniques on paraffin-embedded samples. *Int. J. Cancer*, **64,** 223–228.

WANG XW, YEH H, SCHAEFFER L, ROY R, MONCOLLIN V, EGLY J-M, WANG Z, FRIEDBERG EC, EVANS MK, TAFFE BG, BOHR VA, WEEDA G, HOEIJMAKERS JHJ, FORRESTER K AND HARRIS CC. (1995). p53 associated modulation of TFIIH-associated nucleotide excision repair activity. *Nature Genet.*, **10,** 188–195.

WANG ZW, FORRESTER K, YEH H, FEITELSON MA, GU J-R AND HARRIS CC. (1994). Hepatitis B virus X protein inhibits p53 sequence-specific DNA binding, transcriptional activity, and association with transcription factor ERCC3. *Proc. Natl Acad. Sci. USA*, **91,** 2230–2234.

WRIGHT DK AND MANOS MM. (1990). Sample preparation from paraffin-embedded tissues. In *PCR Protocols – A guide to Methods and Applications*, Innis MA, Gelfand DH, Sninsky JJ and White TJ (eds) pp. 153–158. Academic Press: San Diego, CA, USA.

YIN Y, TAINSKY MA, BISCHOFF FZ, STRONG LC AND WAHL GM. (1992). Wild-type p53 restores cell cycle control and inhibits gene amplification in cells with mutant p53 alleles. *Cell*, **70,** 937–948.

ZHAN Q, BAE I, KASTAN MB AND J FJA. (1994). The p53-dependent $\gamma$-ray response of GADD45. *Cancer*, **54,** 2755–2760.